# The association of genomic alterations with PD‐L1 expression in Chinese patients with EGFR/ALK wild‐type lung adenocarcinoma and potential predictive value of Hippo pathway mutations to immunotherapy

**DOI:** 10.1002/cam4.7038

**Published:** 2024-02-23

**Authors:** Fangfang Liu, Xuemei Zhang, Mengyao Lu, Chun Liu, Xiaodong Zhang, Qian Chu, Yuan Chen, Peng Zhang

**Affiliations:** ^1^ Department of Oncology, Tongji Hospital, Tongji Medical College Huazhong University of Science and Technology Wuhan China; ^2^ Genecast Biotechnology Co., Ltd Wuxi Jiangsu China

**Keywords:** adenocarcinoma, Hippo, immunotherapy, NSCLC, PD‐L1, tumor immune microenvironment

## Abstract

**Background:**

The study focuses on PD‐L1 expression as an essential biomarker for gauging the response of EGFR/ALK wild‐type NSCLC patients to FDA‐approved immune checkpoint inhibitors (ICIs). It aims to explore clinical, molecular, and immune microenvironment characteristics associated with PD‐L1 expression in EGFR/ALK wild‐type lung adenocarcinoma patients eligible for ICI therapy.

**Methods:**

In this retrospective study, tumor samples from 359 Chinese EGFR/ALK wild‐type lung adenocarcinoma patients underwent comprehensive evaluations for PD‐L1 expression and NGS‐targeted sequencing. The investigation encompassed the analysis and comparison of clinical traits, gene mutations, pathways, and immune signatures between two groups categorized by PD‐L1 status: negative (TPS < 1%) and positive (TPS ≥ 1%). Additionally, the study explored the link between genomic changes and outcomes following immunotherapy.

**Results:**

High tumor mutational burden correlated significantly with PD‐L1 positivity in patients with EGFR/ALK wild‐type lung adenocarcinoma. Gene alterations, including TP53, KRAS, and others, were more pronounced in the PD‐L1 positive group. Pathway analysis highlighted higher frequencies of alterations in pathways like RTK/RAS, p53, and Hippo in PD‐L1‐positive patients. The Hippo pathway's relevance was confirmed in separate immunotherapy cohorts, associated with better outcomes. In terms of immune cell infiltration, Hippo mutants exhibited higher levels of CD68^+^PD‐L1^+^ macrophages, CD8^+^ T cells, and CD8^+^PD‐1^−^ T cells.

**Conclusions:**

This study offers insights into genomic features of Chinese EGFR/ALK wild‐type lung adenocarcinoma patients based on PD‐L1 expression. Notably, Hippo pathway alterations were linked to improved immunotherapy outcomes. These findings suggest connections between the Hippo pathway and PD‐L1 expression, warranting further clinical and functional investigations. The research advances our understanding of PD‐L1 expression's genomic context and immunotherapy response in EGFR/ALK wild‐type lung adenocarcinoma.

## 1 INTRODUCTION

In the past decade, new therapies for advanced NSCLC, including tyrosine kinase targeted therapies and immune checkpoint inhibitors (ICIs) based immunotherapy, have led to substantial improvements in clinical effectiveness, including higher objective response rates and prolonged survival.^1^, ^2^ Targeted therapies have been developed against oncogenic drivers in NSCLC, such as mutations in epidermal growth factor receptor (EGFR), and gene rearrangement of anaplastic lymphoma kinase (ALK) and ROS oncogene 1 (ROS1).^1^, ^3^, ^4^ However, these oncogene‐driven tumors exhibit a poor response to ICIs.^5^ Several studies showed an inverse relationship between PD‐L1 expression and EGFR mutations. Moreover, an uninflamed tumor microenvironment is often reported in the context of oncogenic addiction.^6^, ^7^ Accordingly, NCCN guidelines recommend ICIs as the standard care of EGFR or ALK wildtype NSCLC patients. However, it's important to note that not all patients benefit equally from immunotherapy.

In the KEYNOTE‐042 clinical trial, pembrolizumab showed superior clinical outcomes to platinum‐based chemotherapy in patients with metastatic NSCLC exhibiting positive PD‐L1 expression (TPS ≥ 1%).^8^ Subsequent studies have further supported the predictive role of TPS in NSCLC patients without EGFR or ALK genetic alterations. Prior research has established connections between PD‐L1 expression and genetic mutations in driver genes, like EGFR, ALK, TP53, KRAS, STK11, and PTEN,^9^, ^10^, ^11^, ^12^ as well as the activation of various oncogenic pathways, like PI3K/AKT/mTOR, JAK/STAT, and Wnt.^10^, ^13^, ^14^, ^15^, ^16^ However, all these studies included patients with EGFR/ALK alterations who are preferentially treated with targeted therapy rather than immunotherapy. Given this, further characterization of factors associated with PD‐L1 expression specifically in NSCLC patients lacking EGFR or ALK mutations is warranted.

This study analyzed EGFR/ALK wild‐type lung adenocarcinoma patients to explore correlations between gene/pathway alterations and PD‐L1 TPS. We also investigated associations between TPS‐related genomic changes and immunotherapy outcomes, as well as effects on the tumor immune microenvironment. By focusing on EGFR/ALK wild‐type patients, we aimed to elucidate molecular mechanisms and potential biomarkers related to PD‐L1 expression and the response to immunotherapy within this specific and clinically significant subgroup of lung adenocarcinoma patients.

## 2 MATERIALS AND METHODS

### 2.1 Patients and cohorts

In this study, we included patients diagnosed with lung adenocarcinoma who had undergone PD‐L1 testing. To research the targeted population for ICIs, we excluded ALK fusion‐positive or EGFR mutation‐positive patients for whom targeted therapies are recommended in clinical guidelines. The tissues we performed NGS testing simultaneously were limited to the primary tumor. Ethical approval for this study was obtained from the Medical Ethics Committee of Tongji Hospital, Tongji Medical College, Huazhong University of Science and Technology (TJ‐IRB20220971). Prior to enrollment, written informed consent was collected from all patients or their representatives prior to enrollment. All experimental procedures associated with this manuscript were conducted in strict accordance with relevant regulatory guidelines. In total, 359 patients were enrolled, and NGS testing was carried out between February and May 2022 (Table S1). For confirmation of tumor histology and tumor content, all cases underwent a thorough review by pathologists from the Department of Pathology of Tongji Hospital.

To investigate the association between genomic alterations correlated with PD‐L1 expression and immunotherapy outcomes, we utilized three independent immunotherapy cohorts: the *Rizvi.JCO.cohort*, the *Hellmann. Cancer Cell. cohort* and the *Gandara.Nat Med.OAK cohort*, all of which had been published separately.^17^, ^18^, ^19^ At the same time, the patients with ALK fusions or EGFR mutations were also excluded, as shown in Figure S1A; Figure S4A. A new lung adenocarcinoma cohort of 129 patients (TJ‐IRB20220971) with corresponding immunohistochemical staining results was analyzed to explore the effects on tumor‐infiltrating immune cells.

### 2.2 Gene and pathway analysis

Formalin‐fixed paraffin‐embedded (FFPE) tissue specimens of the primary tumors and matched whole blood DNA were collected from each patient for analysis, and targeted gene capture sequencing was performed to assess tumor mutational burden (TMB) using established protocols.^20^, ^21^ DNA was isolated from FFPE tissue specimens with the black PREP FFPE DNA Kit (Analytik Jena, Germany) according to the manufacturers' instructions. FFPE sample‐matched blood lymphocytes were isolated by centrifugation of whole blood at 1600*g* for 10 min at room temperature. Tiangen whole blood DNA kits (Tiangen, Beijing, PRC) were used to extract DNA from the FFPE sample‐matched peripheral blood lymphocytes according to the manufacturer's instructions. Genomic DNA was shewered into 150‐ to 200‐bp fragments with a Covaris M220 Focused‐Ultrasonicator (Covaris, Massachusetts, USA). Fragmented DNA libraries were constructed with a KAPA HTP Library Preparation Kit (Illumina Platform) (KAPA Biosystems, Massachusetts, USA) according to the manufacturer's instructions. DNA libraries were constructed using a custom‐designed panel of 607 genes (see Table S2 for a list of genes), encompassing 1.7 Mb of the entire genome, with a primary focus on genes associated with tumorigenesis. The sequencing depth exceeded 500×, ranging from 552× to 8645×, with a median sequencing depth of 2402×. The signaling pathway analysis was based on a list of critical tumor‐related signaling pathway genes (Table S3). The captured samples underwent sequencing using the Illumina NovaSeq 6000 platform, employing the paired‐end sequencing method. After filtering germline mutations, we selected SNV mutations of all samples according to the following rules: (i) splicing type or exonic region; (ii) depth ≥ 100× and allele frequency ≥ 5%; (iii) allele frequency ≤ 0.2% in the database Exome Aggregation Consortium (ExAC) and Genome Aggregation Database (gnomAD); (iv) mutations without strand bias. Then we got the absolute mutation counts of the tumor samples to calculate TMB with the formula: Absolute mutation counts × 1000,000/Panel exonic base num. TMB was measured in mutations per Mb.

The three validation cohorts used a different DNA library from the NSCLC AD cohort, *Rizvi.JCO.cohort* ^18^ used IMPACT341/410/468, the *Hellmann. Cancer Cell. cohort* ^17^ performed WES in the original study and the *Gandara.Nat Med.OAK cohort* used FoundationOne (F1) CDx NGS assay.^19^ For consistency, we used the DNA library and pathway gene list applied in the NSCLC AD cohort to intersect and apply to the three immunotherapy cohorts.

### 2.3 Immunohistochemical staining

IHC staining was performed to assess the PD‐L1 expression on the surface of tumor cells (TC). FFPE tissue blocks were sliced up into 4‐μm thick sections and stained with anti‐PD‐L1 22C3 primary antibody (1:50, Dako, M3653) using a Ventana GX automated system (Ventana, AZ, USA). PD‐L1 TPS was calculated as the number of PD‐L1 positive tumor cells divided by the total number of tumor cells.

### 2.4 Multiplex immunohistochemical staining

For multiplex immunohistochemical staining, FFPE tissue blocks were sliced up into 4‐μm thick sections. Tissue sections with more than 100 tumor cell counts were considered QC‐qualified for subsequent staining. The immune biomarker panel included CD68 (1:500, Beijing Zhongshan Golden Bridge Biotechnology, ZM0060), CD8 (1:100, Beijing Zhongshan Golden Bridge Biotechnology, ZA0508), PD‐1 (1:50, Beijing Zhongshan Golden Bridge Biotechnology, ZM0381) and PD‐L1 (1:50, Beijing Zhongshan Golden Bridge Biotechnology, ZA‐0629). TSA visualization was performed using an Opal™ 7‐color IHC Kit (PerkinElmer Inc., Boston, MA, USA) according to the manufacturer's instructions and established standard protocols.^21^ The fluorophores used in the experiment were Opal 520 (CD8), Opal 570 (PD‐L1), Opal 650 (CD68), and Opal 690 (PD‐1), with intensity values fixed at 81%. Nuclear counterstaining was performed with DAPI. A PerkinElmer Vectra imaging system (Vectra3.0.5; PerkinElmer, Massachusetts, USA) was used to scan the sections. Regions of interest (ROI) were selected using the Phenochart viewer (Akoya Bioscience) and analysed using inForm Advanced Image Analysis software (InForm 2.3.0; PerkinElmer, Massachusetts, USA). Experienced pathologists circled out multiple tumor and stromal regions on different visions for algorithm training, and then the inForm image analysis software generated a tissue recognition algorithm. The algorithm can be applied to other vision images to recognize tumor and stromal regions in all images. The density of positively stained cells was counted for tumor parenchyma (tumor), distant stroma, and total regions, respectively. In our study, all the specimens were stained with the same panel in the same batch to avoid the batch bias effect.

### 2.5 Statistical analysis

Multi‐group bioinformatics analysis was used to confirm the association of biomarkers with patient outcomes after immunotherapy.^22^, ^23^ Statistical analyses were carried out using R 4.0.4. Survival analysis was conducted using Kaplan–Meier curves, with *p*‐values determined by the log‐rank test. Fisher's exact test was employed to assess statistical heterogeneity, and the Wilcoxon test was used for the comparison of continuous variables. All reported *p*‐values were two‐tailed, and significance was defined as *p* < 0.05. Propensity score matching (PSM) was used to exclude the influence of clinical confounding variables.

## 3 RESULTS

### 3.1 Clinical features and PD‐L1 expression

**FIGURE 1:**
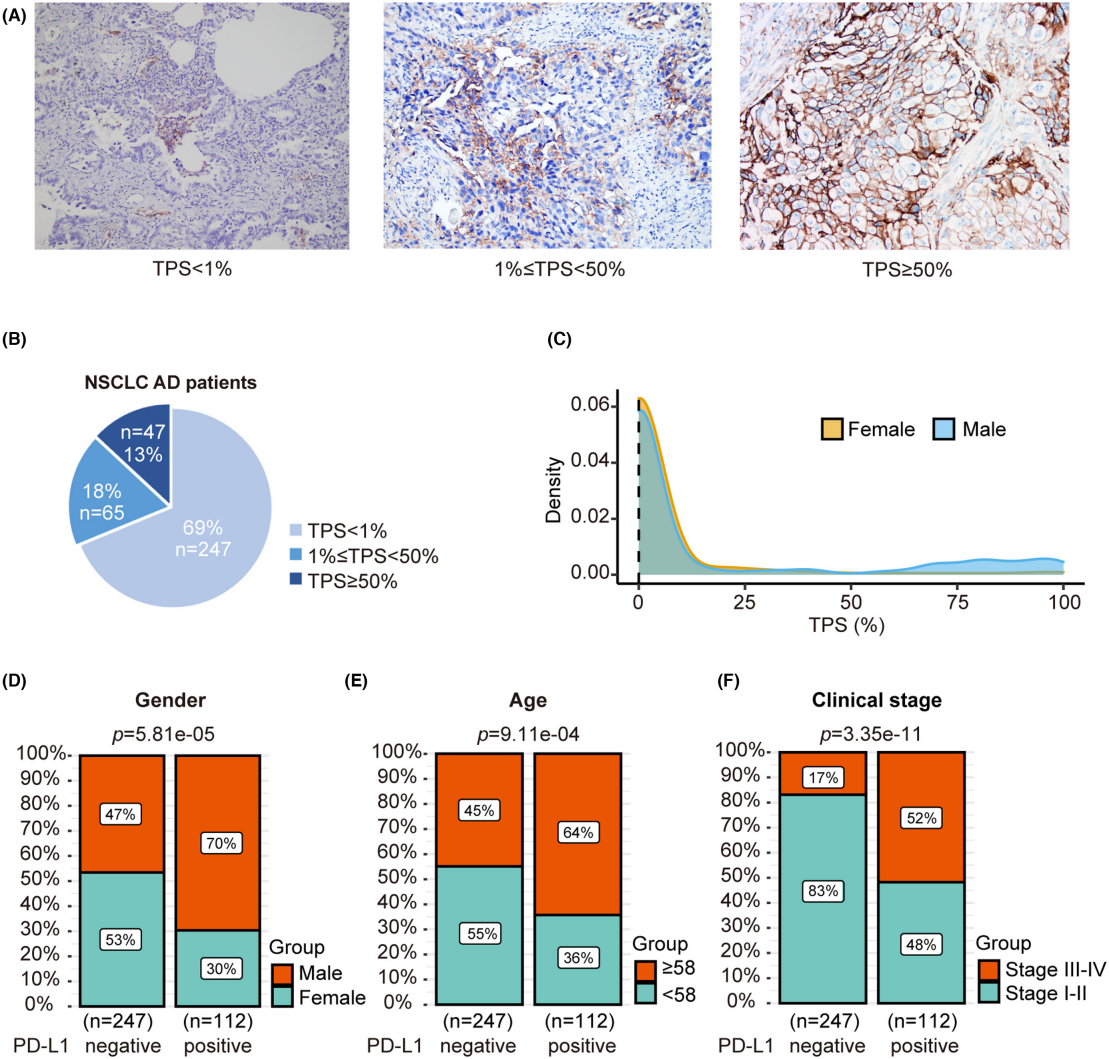
Correlation between PD‐L1 expression and clinical characteristics in the NSCLC AD cohort. (A) PD‐L1 immunohistochemical staining of TPS levels from low to high. (B) PD‐L1 expression in patients with NSCLC AD. (C) Distribution of PD‐L1 expression levels in males and females. (D) Comparison of gender distribution on PD‐L1‐negative and positive subgroups (*p* = 5.81E‐5). (E) Comparison of age distribution on PD‐L1 negative and positive subgroups (*p* = 9.11E‐4). (F) Comparison of clinical stages on PD‐L1 negative and positive subgroups (*p* = 3.35E‐11).

359 NSCLC AD patients who did not possess EGFR mutations or ALK rearrangements were enrolled. Figure 1A shows the immunohistochemical staining of PD‐L1 at different TPS levels. To facilitate our analysis, patients were categorized into two groups based on PD‐L1 levels: those classified as PD‐L1 negative (TPS <1%) and those as PD‐L1 positive (TPS ≥1%). Among the 359 patients, 247 (69%) were PD‐L1 negative, 65 (18%) had a TPS between 1 to 50%, and 47 (13%) had a TPS ≥50% (Figure 1B,C). Of all patients, 193 (66%) were male, and 166 (34%) were female. Notably, the proportion of male patients was significantly higher in the PD‐L1 positive group compared to the PD‐L1 negative group (*p* = 5.81E‐5, Figure 1D). The median age of all lung adenocarcinoma patients was 58 years (range from 25 to 89). Patients over 58 years old were more prevalent in the PD‐L1 positive group (Figure 1E). Moreover, 259 patients (72%) had stage I‐II disease while 100 (28%) had stage III‐IV disease. The proportion of patients with advanced lung adenocarcinoma was significantly higher in the PD‐L1 positive group compared to the negative group (*p* = 3.35E‐11, Figure 1F). The detailed distribution of clinical factors is presented in Table S4.

### 3.2 Genomic mutations associated with PD‐L1 expression

**FIGURE 2:**
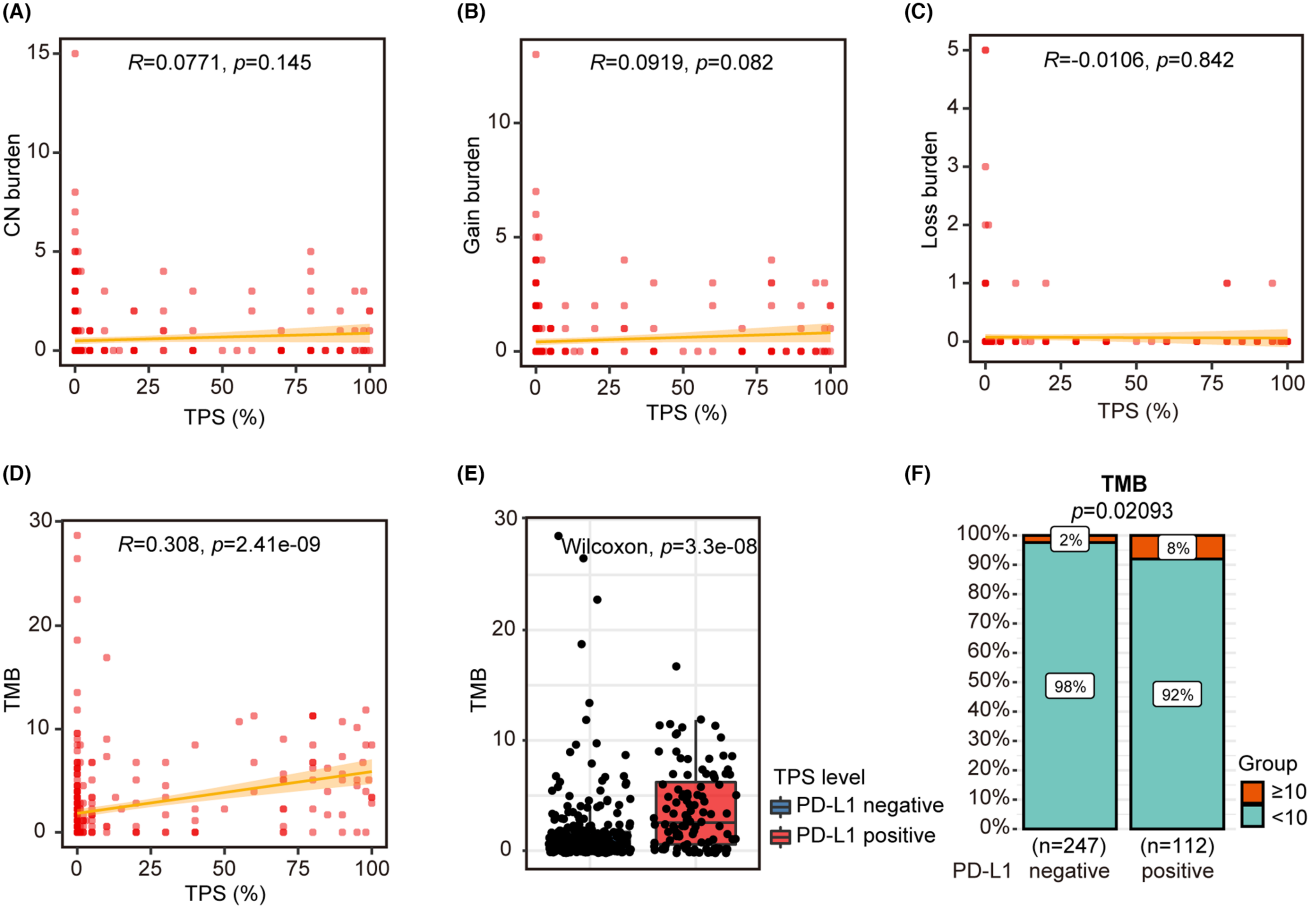
Correlation between PD‐L1 expression and CN burden and TMB in the NSCLC AD cohort. (A) Correlation between copy number burden and TPS. (B) Correlation between copy number gains and TPS. (C) Correlation between copy number losses and TPS. (D) Correlation between the TMB and TPS. (E) Comparison of TMB distribution on PD‐L1 negative and positive subgroups (*p* = 3.3E‐8). (F) Comparison of the proportion of patients with TMB≥10 and TMB < 10 in PD‐L1 positive and negative groups (*p* = 0.02).

In this study cohort, copy number burden (CNburden) was not significantly associated with TPS levels (Figure 2A–C). However, tumor mutational burden (TMB) displayed a noteworthy positive correlation with TPS (*R* = 0.308, *p* = 2.41e‐9, Figure 2D). TMB was obviously higher in PD‐L1‐positive patients versus PD‐L1‐negative patients (Figure 2E), with 8% of PD‐L1‐positive patients having a TMB ≥10 compared to only 2% of PD‐L1‐negative patients (Figure 2F).

**FIGURE 3:**
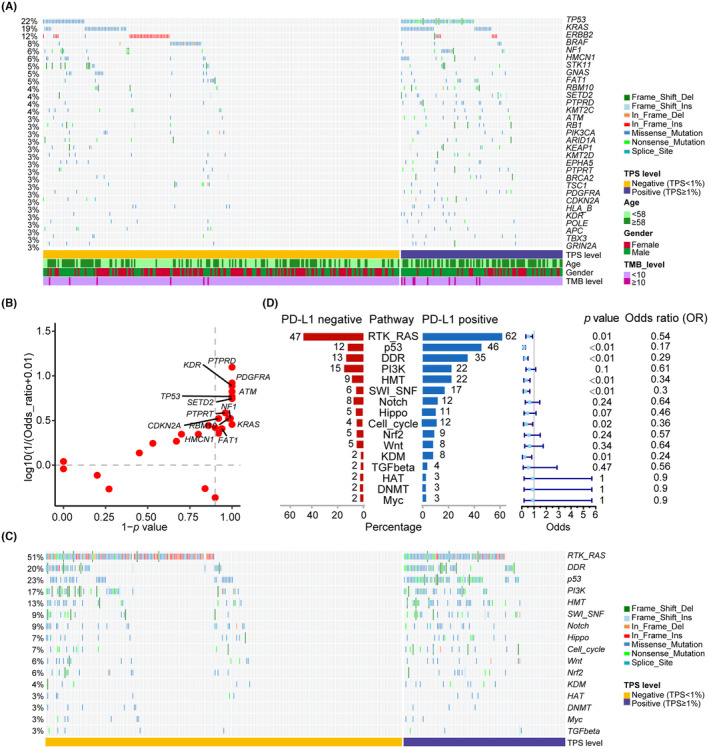
Gene and pathway alterations in different PD‐L1 expression levels in the NSCLC AD cohort. (A) The altered landscape of genes with population frequencies greater than or equal to 3% and in PD‐L1 positive and negative groups. (B) The scatter plot of the altered genes with population frequencies greater than or equal to 3% and in PD‐L1 positive and negative groups. (C) The altered landscape of tumor alterations harboring pathways in PD‐L1 positive and negative groups. (D) Population percentage and forest plot of tumor alterations harboring pathways in PD‐L1 positive and negative groups.

Figure 3A shows mutated genes with a frequency ≥ 3% across all patients. The most commonly altered genes (≥5%) were TP53 (22%), KRAS (19%), ERBB2 (12%), BRAF (8%), NF1 (6%), HMCN1 (6%), STK11 (5%), GNAS (5%) and FAT1 (5%). Thirteen mutated genes were different (*p* < 0.1) between the two groups, all of which were more prevalent in the PD‐L1 positive group. These genes comprised TP53 (46% vs. 12%), KRAS (31% vs. 13%), NF1 (12% vs. 4%), HMCN1 (9% vs. 4%), FAT1 (8% vs. 3%), RBM10 (8% vs. 2%), PTPRD (11% vs. 0.8%), SETD2 (9% vs. 2%), ATM (9% vs. 1%), PTPRT (6% vs. 2%), PDGFRA (7% vs. 0.8%), CDKN2A (5% vs. 2%), and KDR (6% vs. 0.8%) (Figure 3B). Alterations in tumor‐related signaling pathways were more frequent in the PD‐L1 positive group compared to the negative group, including RTK/RAS (62% vs. 47%), p53 (46% vs. 12%), DDR (35% vs. 13%), HMT (22% vs. 9%), SWI/SNF (17% vs. 6%), Cell cycle (12% vs. 4%), and KDM (8% vs. 2%). Furthermore, there was a noticeable trend of enrichment in Hippo pathway alterations in the PD‐L1 positive versus negative group (11% vs. 5%, *p* = 0.07) (Figure 3C,D).

In Figure 1D–F, PD‐L1 expression levels were found to be significantly correlated with gender, age, and clinical stage. In order to control the influence of these confounding variables, propensity score matching (PSM) was performed (Table S5). Based on the matched patient's data (*n* = 226), comparative analyses of pathways among patients with different PD‐L1 expression levels were conducted. The results (Figure S3) indicated that alterations in tumor‐related signaling pathways were more frequent significantly in the PD‐L1 positive group compared to the negative group, including RTK/RAS, p53, DDR, and HMT, which is consistent with the previous results before PSM, and the Hippo pathway alteration remains elevated trend in PD‐L1‐positive patients (*p* = 0.06).

### 3.3 Hippo pathway mutation is associated with immunotherapy efficacy

Due to the small sample sizes for individual gene mutations, we explored the impact of pathway mutations on immunotherapy outcomes in EGFR/ALK wild‐type lung adenocarcinoma patients. We utilized two published cohorts with clinical treatment and outcome data: the *Rizvi.JCO.cohort* (*n* = 132)^18^ and the *Hellmann. Cancer Cell. cohort* (*n* = 49).^17^

**FIGURE 4:**
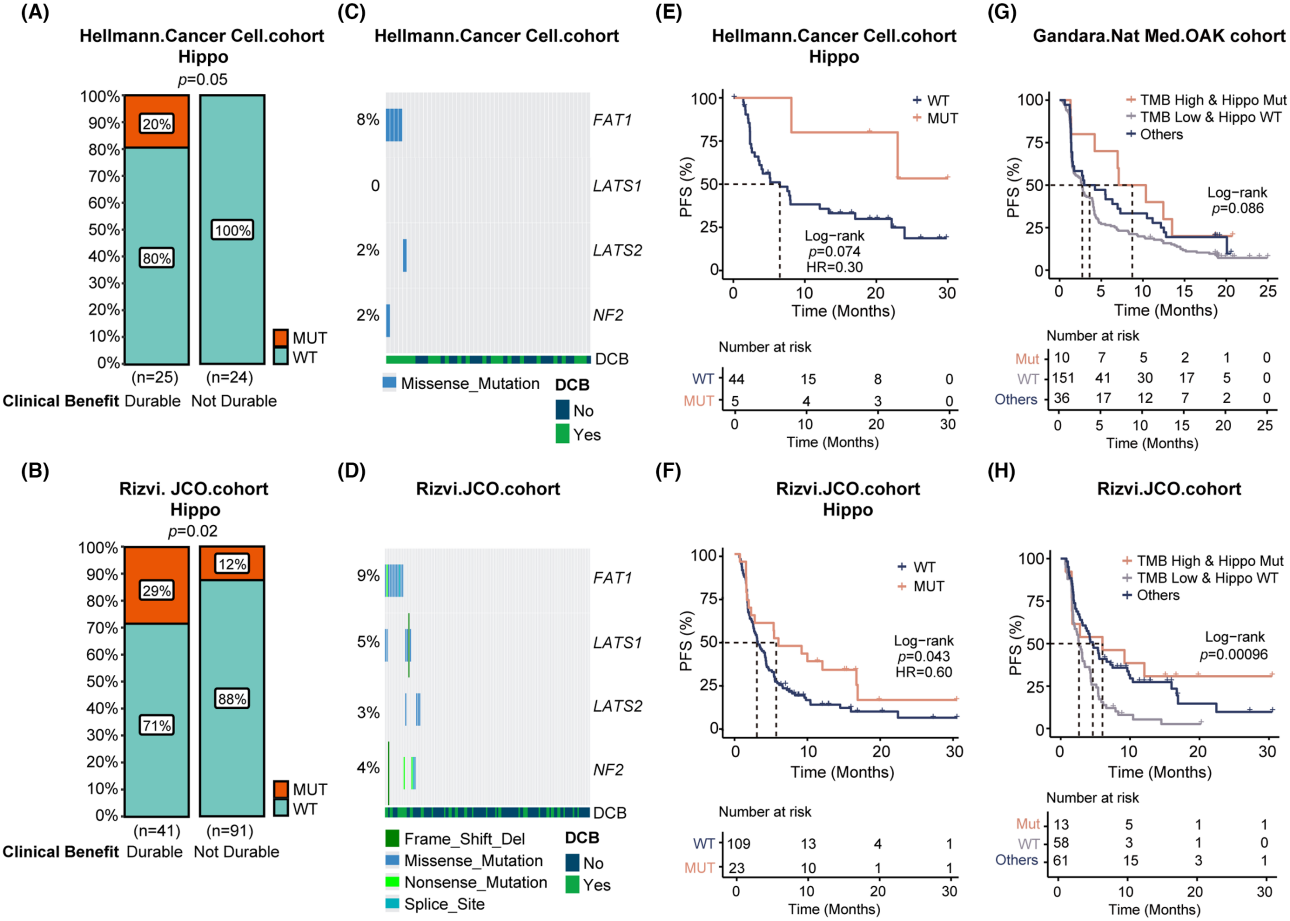
Relationship exploration between Hippo pathway and treatment effect in two external immunotherapy cohorts. Comparison of the proportion of Hippo‐enriched alterations in durable clinical benefit (DCB) and No DCB groups in *Hellmann. Cancer Cell. cohort* (A) and *Rizvi.JCO.cohort* (B). The landscape of the four genes belonging to the Hippo pathway in *Hellmann. Cancer Cell. cohort* (C) *and Rizvi.JCO.cohort* (D). PFS comparison of Hippo pathway mutant and wild‐type groups in *Hellmann. Cancer Cell. cohort* (E) and *Rizvi.JCO.cohort* (F). PFS comparison of TMB high and Hippo mutant group, TMB low and Hippo wild‐type group, and the remaining patients in *Gandara.Nat Med.OAK cohort* (G) and *Rizvi.JCO.cohort* (H).

Firstly, we examined the association of pathway mutations with the durable clinical benefit (DCB) of immunotherapy. Although Cell cycle, DDR, Notch, and Wnt pathway mutations had a higher trend to occur more commonly in patients with DCB in one or both cohorts (*p* < 0.1, black borders, Figure S1B), only Hippo pathway mutations were validated as associated with DCB in both immunotherapy cohorts. In the *Hellmann. Cancer Cell. cohort*, 20% of DCB patients had Hippo mutations versus 0% of non‐DCB patients (*p* = 0.05). And in the *Rizvi.JCO.cohort*, 29% of DCB patients had Hippo pathway mutations versus 12% of non‐DCB patients (*p* = 0.02, Figure 4A,B). Among the panels used for NGS analysis in this study, four genes belong to the Hippo pathway, namely FAT1, LATS1, LATS2, and NF2. FAT1 mutations were most frequent in both Chinese and Western cohorts (Figure 4C,D; Figure S2). Due to the small Hellmann cohort size, LATS1 mutations were not observed. Consistent with DCB, Hippo pathway mutations were associated with superior progression‐free survival (PFS) in the *Rizvi.JCO.cohort* (*p* = 0.043) and *Hellmann. Cancer Cell. cohort* (*p* = 0.074), suggesting the Hippo pathway may predict immunotherapy response in lung adenocarcinoma (Figure 4E,F; Figure S1C). Although the lists of Hippo pathway genes included in the panels used by several cohorts are not entirely consistent, they all encompass key mutated genes such as LATS1, LATS2, NF1, and FAT1 (Figure 4C,D). The correlation between Hippo pathway mutations in tumor tissues and the efficacy of immunotherapy could be validated in the *Hellmann. Cancer Cell. cohort* and *Rizvi.JCO.cohort* indicating the Hippo pathway plays an important role in regulating cancer cell fate and its communication with TIME.

We then explored the effect of Hippo alterations combined with TMB levels on the survival of patients after immunotherapy. A stratified analysis was performed on *Rizvi.JCO.cohort* ^18^ and *Gandara.Nat Med.OAK cohort* ^19^ (Figure S4A) according to the TMB cutoff values reported in the original article. Although in the *Gandara.Nat Med.OAK cohort*, Hippo mutations were not significantly associated with better survival (Figure S4B), patients with both high TMB and Hippo alterations had the best survival, and patients with low TMB and Hippo wild‐type had the worst survival, whereas others had survival curves in between (*p* = 0.086). Consistent results were also obtained for the *Rizvi.JCO.cohort* (*p* < 0.001) (Figure 4G,H). *Hellmann. Cancer Cell. cohort* was too small for further TMB stratified analysis. Thus, Hippo mutations are associated with better survival in NSCLC adenocarcinoma patients receiving immunotherapy, but this may be influenced by TMB levels.

### 3.4 Hippo pathway mutation impacts on tumor immune microenvironment

**FIGURE 5:**
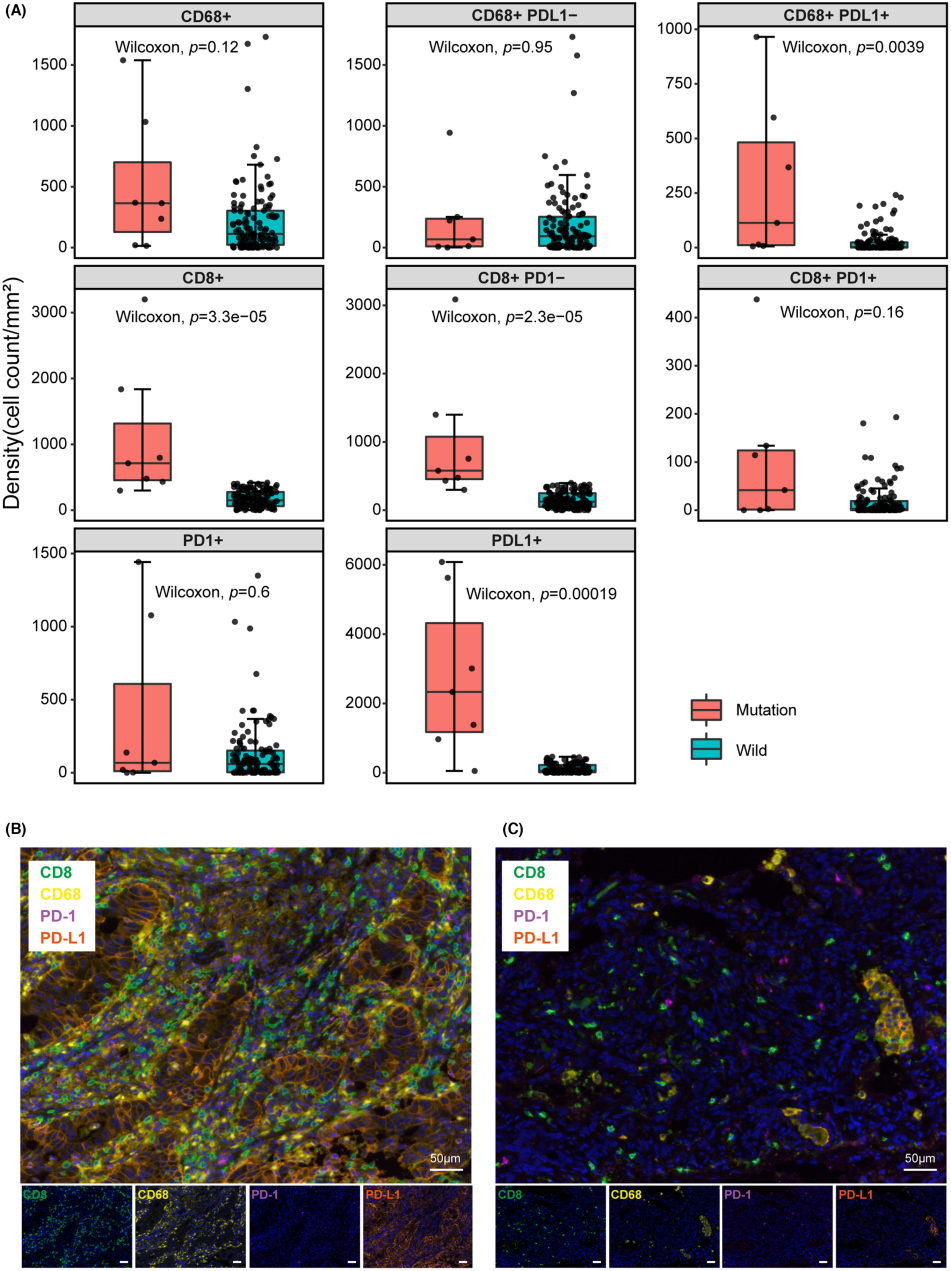
The association between PD‐L1 expression levels and tumor‐infiltrating lymphocytes (TILs) or immune checkpoints in a new lung adenocarcinoma cohort. (A) Comparison of PD‐1^+^, PD‐L1^+^ cell density and CD68^+^, CD68^+^PD‐L1^−^, and CD68^+^PD‐L1^+^macrophage, CD8^+^, CD8^+^PD‐1^−^, and CD8^+^PD‐1^+^ T cell density between the Hippo mutant and Hippo wild‐type groups in the entire region. (B) Immunohistochemical staining and merge Images of CD8, CD68, PD‐1, and PD‐L1 in tissues from a patient with Hippo mutation. (C) Immunohistochemical staining and merge Images of CD8, CD68, PD‐1, and PD‐L1 in tissues from a patient with Hippo wild type. Bar = 50 μm.

Building upon our earlier findings, we investigated the influence of the Hippo pathway alteration on the immune microenvironment. Among 129 patients with immunohistochemistry data, seven patients had Hippo pathway gene mutations (Hippo mutant group) while 122 were Hippo wild‐type. In the total area, we observed higher densities of PD‐L1^+^ cells (*p* = 0.00019) and CD8^+^ T cells (*p* = 3.3e‐05) in the Hippo mutant group, with a significant trend. However, no significant difference was noted in the density of PD‐1+ cells (*p* = 0.6). Notably, the density of CD68^+^PD‐L1^+^ macrophage cells in the total area was substantially greater in the Hippo mutant group compared to the Hippo wild‐type group (*p* = 0.0039). In contrast, there was no significant difference in the density of CD68^+^PD‐L1^−^ macrophages (*p* = 0.95). Furthermore, the density of CD8^+^PD‐1^−^ T cells was significantly elevated in the Hippo mutant group in the total area (*p* = 2.3e‐05), while the density of CD8^+^PD‐1^+^ T cells did not exhibit an obvious difference (*p* = 0.16) (Figure 5A). The immunohistochemical staining images are shown for Hippo mutant (Figure 5B) and Hippo wild‐type (Figure 5C) patients.

## 4 DISCUSSION

Although some studies have indicated either a positive or no correlation between PD‐L1 and EGFR mutations,^24^ clinical evidence demonstrates the lack of efficacy of ICI monotherapy in TKI‐naive, PD‐L1‐positive, and EGFR‐mutant patients with advanced NSCLC.^25^ Independent studies have consistently revealed a strong association between PD‐L1 positivity and the absence of EGFR mutations in NSCLC patients.^26^, ^27^, ^28^ Moreover, the combination of ICI and TKI resulted in an increased incidence of adverse events without evident benefits.^29^ Therefore, our research focuses on patients with EGFR/ALK wild‐type lung adenocarcinoma who may benefit from ICI treatment. The PD‐L1 IHC companion diagnostic has been acknowledged for NSCLC patients undergoing ICI treatment, and positive PD‐L1 expression is associated with a higher response rate and prolonged survival during ICI therapy.^30^, ^31^ However, PD‐L1's utility as a biomarker is hindered by instability and heterogeneity.^32^, ^33^ Hence, we attempt to explore the association between genomic alterations and PD‐L1 expression in the hope of unveiling novel insights for the treatment of this patient subgroup.

Adam J. Schoenfeld and colleagues investigated PD‐L1 expression in 1586 lung adenocarcinoma patients,^10^ categorizing them into high (≥50%), low (1%–49%), and negative (<1%) expression groups, with a focus on discerning differences between the high and negative expression groups. While acknowledging the 18% with low PD‐L1 expression, our study primarily compares PD‐L1 negative (<1%) and positive (≥1%) groups to identify distinctions, broadening the scope of potential biomarkers, which is consistent with the KEYNOTE‐042 trial, it expands the population of patients benefiting from pembrolizumab monotherapy to TPS≥1%.^8^ Approximately 30% of our lung adenocarcinoma cohort exhibited positive PD‐L1 expression, consistent with previous reported.^10^ Male gender, advanced age, and high TMB levels were remarkably correlated with positive PD‐L1 expression, aligning with established trends.^16^ At the level of individual gene mutations, our study unveiled that KRAS and TP53 mutations were more prevalent in PD‐L1‐positive patients, corroborating findings in the broader NSCLC population. RAS signaling/TP53 upregulates tumor cell PD‐L1 expression through increases in transcription of PD‐L1 and PD‐L1 mRNA stability.^34^, ^35^, ^36^ Intriguingly, we identified novel molecular alterations linked to varying PD‐L1 expression, including PTPRD phosphatase, RNA‐binding protein RBM10, and Hippo pathway members NF1 and FAT1. In addition to DDR, p53, RTK/RAS, and cell cycle pathways, pathways that mediate chromatin remodeling such as HMT, SWI_SNF, and KDM had higher mutational frequency in the PD‐L1‐positive group. This supports previous findings that histone deacetylase (HDAC1/2),^37^ histone methyltransferase EZH2,^38^ KMT2A^39^ regulate the transcription of PD‐L1. Collectively, these findings suggest that genomic mutations exert specific effects on PD‐L1 expression within the EGFR/ALK wild‐type population.

Our study identified the Hippo pathway as a key predictor of sustained response to immune checkpoint inhibitor (ICI) therapy in EGFR/ALK wild‐type cohorts. Mutations in FAT1, a gene of Hippo pathway, were associated with higher durable clinical benefit and significantly improved survival outcomes than wild‐type NSCLC patients, same results were also confirmed in melanoma patients and a pan‐cancer cohort.^40^, ^41^, ^42^ It suggested the possible generalizability of the study findings to other populations and clinical settings. In contrast to our findings, Talb et al. observed significant activation of the Hippo pathway in pembrolizumab‐refractory patients, potentially influenced by a small cohort size (*n* = 19), ethnic variations, patient characteristics (including 3 squamous cell carcinomas), and methodological disparities.^43^ Further investigations into the role of the Hippo pathway in the context of PD‐1/immunotherapy are merited. Hippo pathway plays key roles across biological processes and diseases,^44^ Dysregulation of the Hippo signaling pathway, a common occurrence in human malignancies, involves YAP1 activation.^45^, ^46^ Loss of function or genetic mutations in Hippo kinases like NF2 and LATS1/2 leads to the activation of YAP, fostering positive interactions with other pathways and driving tumor progression.^47^, ^48^, ^49^ The Hippo pathway's role in immune regulation is complex. In NSCLC tissues, co‐expression of YAP and PD‐L1 is observed. YAP influences PD‐L1 at the genetic level by attaching to the PD‐L1 enhancer region to escape immune surveillance.^50^ The immune cells infiltration analysis also indicates that Hippo pathway mutation affects the CD8^+^ T cells infiltration, providing the “hot” environments for ICIs therapy. Guan et al. found that LATS1/2 knockdown in tumor cells enhanced immunogenicity and inhibited cell proliferation, highlighting the “oncogenic” role of LATS1/2 suppressing immunogenicity. This reveals an “oncogenic” phenotype of LATS1/2 that inhibits immunogenicity and identifies them as potential immunotherapy targets.^51^ NSCLC patients with FAT1 mutation exhibited increased M1 macrophage infiltration, decreased M2 macrophage and T‐regulatory cell infiltration, along significantly elevated activated dendritic cells in FAT1‐mutated LUAD patients.^42^, ^52^ This supports the notion of a “hot” tumor microenvironment in tumors with Hippo pathway mutation. Several studies indicated Verteporfin could suppress the formation of YAP/TEAD complex,^53^, ^54^ and a peptide mimicking function of VGLL4 could disrupt YAP/TEAD interaction.^55^, ^56^ Given these findings, it is evident that the Hippo pathway represents a promising target for the development of anti‐cancer drugs, particularly for combinational therapy aimed at overcoming drug resistance or achieving higher efficacy.

The relatively short follow‐up limits our ability to assess long‐term treatment responses and overall survival benefits associated with observed PD‐L1 expression patterns. Further exploration is needed to determine the generalizability of our study findings to diverse populations and clinical settings.

## 5 CONCLUSION

To sum up, our research offers insights into the genomic landscape of Chinese lung adenocarcinoma patients without EGFR mutations or ALK fusions potentially treated with ICIs. We have specifically highlighted the significance of the Hippo pathway and its connection to the effectiveness of immunotherapy in two previously studied cohorts. Importantly, we have also established a compelling association between Hippo pathway mutations and the infiltration of CD68^+^PD‐L1^+^ macrophages, CD8^+^ T cells, and CD8^+^PD‐1^−^ T cells. These findings hold promise for advancing the treatment of EGFR/ALK wild‐type lung adenocarcinoma patients who are being considered for ICI therapy.

## AUTHOR CONTRIBUTIONS

**Fangfang Liu:** Conceptualization (equal); data curation (equal); formal analysis (equal); writing – original draft (lead). **Xuemei Zhang:** Writing – original draft (supporting). **Mengyao Lu:** Formal analysis (supporting); methodology (equal). **Chun Liu:** Data curation (supporting); formal analysis (supporting); methodology (equal); software (equal). **Xiaodong Zhang:** Formal analysis (supporting); methodology (equal); software (supporting). **Qian Chu:** Writing – review and editing (equal). **Yuan Chen:** Writing – review and editing (equal). **Peng Zhang:** Conceptualization (lead); funding acquisition (equal); project administration (lead).

## FUNDING INFORMATION

This work was funded by the National Natural Science Foundation of China (No. 81974483 and 82072589).

## CONFLICT OF INTEREST STATEMENT

The authors declare no conflicts of interest.

## ETHICS APPROVAL

This study received approval from the Medical Ethics Committee of Tongji Hospital, Tongji Medical College, Huazhong University of Science and Technology (Approval No. TJ‐IRB20220971). Informed consent was obtained from all study participants or their representatives prior to enrollment. All experimental procedures adhered to relevant guidelines and regulations.

## CONSENT FOR PUBLICATION

Not applicable.

## Supporting information


Figure S1.

Figure S2.

Figure S3.

Figure S4.

Table S1.

Table S2.

Table S3.

Table S4.

Table S5.

## Data Availability

The data and materials, including next‐generation sequencing and immune histochemistry data, are available upon reasonable request.

## References

[1] Saber A , van der Wekken A , Hiltermann TJN , Kok K , van den Berg A , Groen HJM . Genomic aberrations guiding treatment of non‐small cell lung cancer patients. Cancer Treat Commun. 2015;4:23‐33.

[2] Ogunleye F , Blankenship L , Millisor V , Anderson J , Jaiyesimi I . Programmed cell death‐1/programmed cell death ligand‐1(PD‐1/PD‐L1) inhibitors, heralding a new era of immunotherapy in the management of advanced non‐small cell lung cancer (NSCLC). Cancer Treat Res Commun. 2017;12:6‐13.

[3] Singal G , Miller PG , Agarwala V , et al. Association of patient characteristics and tumor genomics with clinical outcomes among patients with non‐small cell lung cancer using a clinicogenomic database. JAMA. 2019;321:1391‐1399. 30964529 10.1001/jama.2019.3241 PMC6459115

[4] Herbst RS , Morgensztern D , Boshoff C . The biology and management of non‐small cell lung cancer. Nature. 2018;553:446‐454. 29364287 10.1038/nature25183

[5] Mazieres J , Drilon A , Lusque A , et al. Immune checkpoint inhibitors for patients with advanced lung cancer and oncogenic driver alterations: results from the IMMUNOTARGET registry. Ann Oncol. 2019;30:1321‐1328. 31125062 10.1093/annonc/mdz167 PMC7389252

[6] Soo RA , Lim SM , Syn NL , et al. Immune checkpoint inhibitors in epidermal growth factor receptor mutan t non‐small cell lung cancer: current controversies and future directi ons. Lung Cancer (Amsterdam, Netherlands). 2018;115:12‐20. 29290252 10.1016/j.lungcan.2017.11.009

[7] Dong Z‐Y , Zhang J‐T , Liu S‐Y , et al. EGFR mutation correlates with uninflamed phenotype and weak immunogeni city, causing impaired response to PD‐1 blockade in non‐small cell lun g cancer. Onco Targets Ther. 2017;6:e1356145. 10.1080/2162402X.2017.1356145 PMC5674946 29147605

[8] Mok TSK , Wu YL , Kudaba I , et al. Pembrolizumab versus chemotherapy for previously untreated, PD‐L1‐expressing, locally advanced or metastatic non‐small‐cell lung cancer (KEYNOTE‐042): a randomised, open‐label, controlled, phase 3 trial. Lancet. 2019;393:1819‐1830. 30955977 10.1016/S0140-6736(18)32409-7

[9] Chen J , Jiang CC , Jin L , Zhang XD . Regulation of PD‐L1: a novel role of pro‐survival signalling in cancer. Ann Oncol. 2016;27:409‐416. 26681673 10.1093/annonc/mdv615

[10] Schoenfeld AJ , Rizvi H , Bandlamudi C , et al. Clinical and molecular correlates of PD‐L1 expression in patients with lung adenocarcinomas. Ann Oncol. 2020;31:599‐608. 32178965 10.1016/j.annonc.2020.01.065 PMC7523592

[11] Lamberti G , Spurr LF , Li Y , et al. Clinicopathological and genomic correlates of programmed cell death ligand 1 (PD‐L1) expression in nonsquamous non‐small‐cell lung cancer. Ann Oncol. 2020;31:807‐814. 32171752 10.1016/j.annonc.2020.02.017

[12] Isomoto K , Haratani K , Hayashi H , et al. Impact of EGFR‐TKI treatment on the tumor immune microenvironment in EGFR mutation‐positive non‐small cell lung cancer. Clin Cancer Res. 2020;26:2037‐2046. 31937613 10.1158/1078-0432.CCR-19-2027

[13] Song TL , Nairismagi ML , Laurensia Y , et al. Oncogenic activation of the STAT3 pathway drives PD‐L1 expression in natural killer/T‐cell lymphoma. Blood. 2018;132:1146‐1158. 30054295 10.1182/blood-2018-01-829424 PMC6148343

[14] Rennier K , Shin WJ , Krug E , Virdi G , Pachynski RK . Chemerin reactivates PTEN and suppresses PD‐L1 in tumor cells via modulation of a novel CMKLR1‐mediated signaling cascade. Clin Cancer Res. 2020;26:5019‐5035. 32605911 10.1158/1078-0432.CCR-19-4245

[15] Atefi M , Avramis E , Lassen A , et al. Effects of MAPK and PI3K pathways on PD‐L1 expression in melanoma. Clin Cancer Res. 2014;20:3446‐3457. 24812408 10.1158/1078-0432.CCR-13-2797 PMC4079734

[16] Li K , Liu J , Wu L , et al. Genomic correlates of programmed cell death ligand 1 (PD‐L1) expression in Chinese lung adenocarcinoma patients. Cancer Cell Int. 2022;22:138. 35346207 10.1186/s12935-022-02488-z PMC8962080

[17] Hellmann MD , Nathanson T , Rizvi H , et al. Genomic features of response to combination immunotherapy in patients with advanced non‐small‐cell lung cancer. Cancer Cell. 2018;33:843‐852. 29657128 10.1016/j.ccell.2018.03.018 PMC5953836

[18] Rizvi H , Sanchez‐Vega F , La K , et al. Molecular determinants of response to anti‐programmed cell death (PD)‐1 and anti‐programmed death‐ligand 1 (PD‐L1) blockade in patients with non‐small‐cell lung cancer profiled with targeted next‐generation sequencing. J Clin Oncol. 2018;36:633‐641. 29337640 10.1200/JCO.2017.75.3384 PMC6075848

[19] Gandara DR , Paul SM , Kowanetz M , et al. Blood‐based tumor mutational burden as a predictor of clinical benefit in non‐small‐cell lung cancer patients treated with atezolizumab. Nat Med. 2018;24:1441‐1448. 30082870 10.1038/s41591-018-0134-3

[20] Chen Y , Liu Q , Chen Z , et al. PD‐L1 expression and tumor mutational burden status for prediction of response to chemotherapy and targeted therapy in non‐small cell lung cancer. J Exp Clin Cancer Res. 2019;38:193. 31088500 10.1186/s13046-019-1192-1 PMC6518807

[21] Zhang L , Chen Y , Wang H , et al. Massive PD‐L1 and CD8 double positive TILs characterize an immunosuppressive microenvironment with high mutational burden in lung cancer. J Immunother Cancer. 2021;9:e002356. 34140315 10.1136/jitc-2021-002356 PMC8212410

[22] Dang HH , Ta HDK , Nguyen TTT , et al. Prospective role and immunotherapeutic targets of sideroflexin protein family in lung adenocarcinoma: evidence from bioinformatics validation. Funct Integr Genomics. 2022;22:1057‐1072. 35851932 10.1007/s10142-022-00883-3

[23] Tran TO , Vo TH , Lam LHT , et al. ALDH2 as a potential stem cell‐related biomarker in lung adenocarcinoma: comprehensive multi‐omics analysis. Comput Struct Biotechnol J. 2023;21:1921‐1929. 36936815 10.1016/j.csbj.2023.02.045 PMC10018390

[24] Zhang Y , Wang L , Li Y , et al. Protein expression of programmed death 1 ligand 1 and ligand 2 independently predict poor prognosis in surgically resected lung adenocarcinoma. Onco Targets Ther. 2014;7:567‐573. 24748806 10.2147/OTT.S59959 PMC3990506

[25] Lisberg A , Cummings A , Goldman JW , et al. A phase II study of Pembrolizumab in EGFR‐mutant, PD‐L1+, tyrosine kinase inhibitor Naïve patients with advanced NSCLC. J Thorac Oncol. 2018;13:1138‐1145. 29874546 10.1016/j.jtho.2018.03.035 PMC6063769

[26] Kojima K , Sakamoto T , Kasai T , Kagawa T , Yoon H , Atagi S . PD‐L1 expression as a predictor of postoperative recurrence and the association between the PD‐L1 expression and EGFR mutations in NSCLC. Sci Rep. 2021;11:17522. 34471191 10.1038/s41598-021-96938-9 PMC8410871

[27] Inamura K , Yokouchi Y , Sakakibara R , et al. Relationship of tumor PD‐L1 expression with EGFR wild‐type status and poor prognosis in lung adenocarcinoma. Jpn J Clin Oncol. 2016;46:935‐941. 27511990 10.1093/jjco/hyw087

[28] Alwithenani A , Bethune D , Castonguay M , et al. Profiling non‐small cell lung cancer reveals that PD‐L1 is associated with wild type EGFR and vascular invasion, and immunohistochemistry quantification of PD‐L1 correlates weakly with RT‐qPCR. PLoS One. 2021;16:e0251080. 33956842 10.1371/journal.pone.0251080 PMC8101740

[29] Yang JC , Shepherd FA , Kim DW , et al. Osimertinib plus durvalumab versus osimertinib monotherapy in EGFR T790M‐positive NSCLC following previous EGFR TKI therapy: CAURAL brief report. J Thorac Oncol. 2019;14:933‐939. 30763730 10.1016/j.jtho.2019.02.001

[30] Garon EB , Hellmann MD , Rizvi NA , et al. Five‐year overall survival for patients with advanced non–small‐cell lung cancer treated with pembrolizumab: results from the phase I KEYNOTE‐001 study. J Clin Oncol. 2019;37:2518‐2527. 31154919 10.1200/JCO.19.00934 PMC6768611

[31] Gadgeel S , Rodríguez‐Abreu D , Speranza G , et al. Updated analysis from KEYNOTE‐189: pembrolizumab or placebo plus pemetrexed and platinum for previously untreated metastatic nonsquamous non‐small‐cell lung cancer. J Clin Oncol. 2020;38:1505‐1517. 32150489 10.1200/JCO.19.03136

[32] Davis AA , Patel VG . The role of PD‐L1 expression as a predictive biomarker: an analysis of all US Food and Drug Administration (FDA) approvals of immune checkpoint inhibitors. J Immunother Cancer. 2019;7:278. 31655605 10.1186/s40425-019-0768-9 PMC6815032

[33] Patel SP , Kurzrock R . PD‐L1 expression as a predictive biomarker in cancer immunotherapy. Mol Cancer Ther. 2015;14:847‐856. 25695955 10.1158/1535-7163.MCT-14-0983

[34] Coelho MA , de Carné Trécesson S , Rana S , et al. Oncogenic RAS signaling promotes tumor immunoresistance by stabilizing PD‐L1 mRNA. Immunity. 2017;47:1083‐1099. 29246442 10.1016/j.immuni.2017.11.016 PMC5746170

[35] Yu J , Ling S , Hong J , et al. TP53/mTORC1‐mediated bidirectional regulation of PD‐L1 modulates immune evasion in hepatocellular carcinoma. J Immunother Cancer. 2023;11:e007479. 38030304 10.1136/jitc-2023-007479 PMC10689408

[36] Dong ZY , Zhong WZ , Zhang XC , et al. Potential predictive value of TP53 and KRAS mutation status for response to PD‐1 blockade immunotherapy in lung adenocarcinoma. Clin Cancer Res. 2017;23:3012‐3024. 28039262 10.1158/1078-0432.CCR-16-2554

[37] Shen Y , Liu L , Wang M , et al. TET2 inhibits PD‐L1 gene expression in breast cancer cells through histone deacetylation. Cancers. 2021;13:2207. 34064441 10.3390/cancers13092207 PMC8125390

[38] Xiao G , Jin LL , Liu CQ , et al. EZH2 negatively regulates PD‐L1 expression in hepatocellular carcinoma. J Immunother Cancer. 2019;7:300. 31727135 10.1186/s40425-019-0784-9 PMC6854886

[39] Lu C , Paschall AV , Shi H , et al. The MLL1‐H3K4me3 axis‐mediated PD‐L1 expression and pancreatic cancer immune evasion. J Natl Cancer Inst. 2017;109:djw283. 28131992 10.1093/jnci/djw283 PMC5291187

[40] Fang W , Ma Y , Yin JC , et al. Comprehensive genomic profiling identifies novel genetic predictors of response to anti‐PD‐(L)1 therapies in non‐small cell lung cancer. Clin Cancer Res. 2019;25:5015‐5026. 31085721 10.1158/1078-0432.CCR-19-0585

[41] Zhu G , Ren D , Lei X , et al. Mutations associated with No durable clinical benefit to immune checkpoint blockade in non‐S‐cell lung cancer. Cancers (Basel). 2021;13:1397. 33808631 10.3390/cancers13061397 PMC8003499

[42] Zhang W , Tang Y , Guo Y , et al. Favorable immune checkpoint inhibitor outcome of patients with melanoma and NSCLC harboring FAT1 mutations. NPJ Precis Oncol. 2022;6:46. 35739249 10.1038/s41698-022-00292-6 PMC9226130

[43] Talb J , Takam Kamga P , Mayenga M , et al. Gene expression profile of high PD‐L1 non‐small cell lung cancers refractory to pembrolizumab. Cancer Immunol Immunother. 2022;71:2791‐2799. 35435450 10.1007/s00262-022-03206-4 PMC10992407

[44] Dey A , Varelas X , Guan KL . Targeting the hippo pathway in cancer, fibrosis, wound healing and regenerative medicine. Nat Rev Drug Discov. 2020;19:480‐494. 32555376 10.1038/s41573-020-0070-z PMC7880238

[45] Gujral TS , Kirschner MW . Hippo pathway mediates resistance to cytotoxic drugs. Proc Natl Acad Sci USA. 2017;114:E3729‐e3738. 28416665 10.1073/pnas.1703096114 PMC5422801

[46] Lee JE , Park HS , Lee D , et al. Hippo pathway effector YAP inhibition restores the sensitivity of EGFR‐TKI in lung adenocarcinoma having primary or acquired EGFR‐TKI resistance. Biochem Biophys Res Commun. 2016;474:154‐160. 27105908 10.1016/j.bbrc.2016.04.089

[47] Martin D , Degese MS , Vitale‐Cross L , et al. Assembly and activation of the hippo signalome by FAT1 tumor suppressor. Nat Commun. 2018;9:2372. 29985391 10.1038/s41467-018-04590-1 PMC6037762

[48] White SM , Avantaggiati ML , Nemazanyy I , et al. YAP/TAZ inhibition induces metabolic and signaling rewiring resulting in targetable vulnerabilities in NF2‐deficient tumor cells. Dev Cell. 2019;49:425‐443. 31063758 10.1016/j.devcel.2019.04.014 PMC6524954

[49] van Rensburg HJ , Yang X . The roles of the hippo pathway in cancer metastasis. Cell Signal. 2016;28:1761‐1772. 27519476 10.1016/j.cellsig.2016.08.004

[50] Miao J , Hsu PC , Yang YL , et al. YAP regulates PD‐L1 expression in human NSCLC cells. Oncotarget. 2017;8:114576‐114587. 29383103 10.18632/oncotarget.23051 PMC5777715

[51] Moroishi T , Hayashi T , Pan WW , et al. The hippo pathway kinases LATS1/2 suppress cancer immunity. Cell. 2016;167:1525‐1539. 27912060 10.1016/j.cell.2016.11.005 PMC5512418

[52] Feng Z , Yin Y , Liu B , et al. Prognostic and immunological role of FAT family genes in non‐small cell lung cancer. Cancer Control. 2022;29:10732748221076682. 35212236 10.1177/10732748221076682 PMC8891876

[53] Liu‐Chittenden Y , Huang B , Shim JS , et al. Genetic and pharmacological disruption of the TEAD‐YAP complex suppresses the oncogenic activity of YAP. Genes Dev. 2012;26:1300‐1305. 22677547 10.1101/gad.192856.112 PMC3387657

[54] Ye S , Eisinger‐Mathason TS . Targeting the hippo pathway: clinical implications and therapeutics. Pharmacol Res. 2016;103:270‐278. 26678601 10.1016/j.phrs.2015.11.025

[55] Zhang W , Gao Y , Li P , et al. VGLL4 functions as a new tumor suppressor in lung cancer by negatively regulating the YAP‐TEAD transcriptional complex. Cell Res. 2014;24:331‐343. 24458094 10.1038/cr.2014.10 PMC3945886

[56] Jiao S , Wang H , Shi Z , et al. A peptide mimicking VGLL4 function acts as a YAP antagonist therapy against gastric cancer. Cancer Cell. 2014;25:166‐180. 24525233 10.1016/j.ccr.2014.01.010

