# Supplementary figures and images for: The association of genomic alterations with PD‐L1 expression in Chinese patients with EGFR/ALK wild‐type lung adenocarcinoma and potential predictive value of Hippo pathway mutations to immunotherapy
Source: Cancer Med. 2024 Feb 23;13(3):e7038. doi: 10.1002/cam4.7038 (PMC10891359; doi:10.1002/cam4.7038)

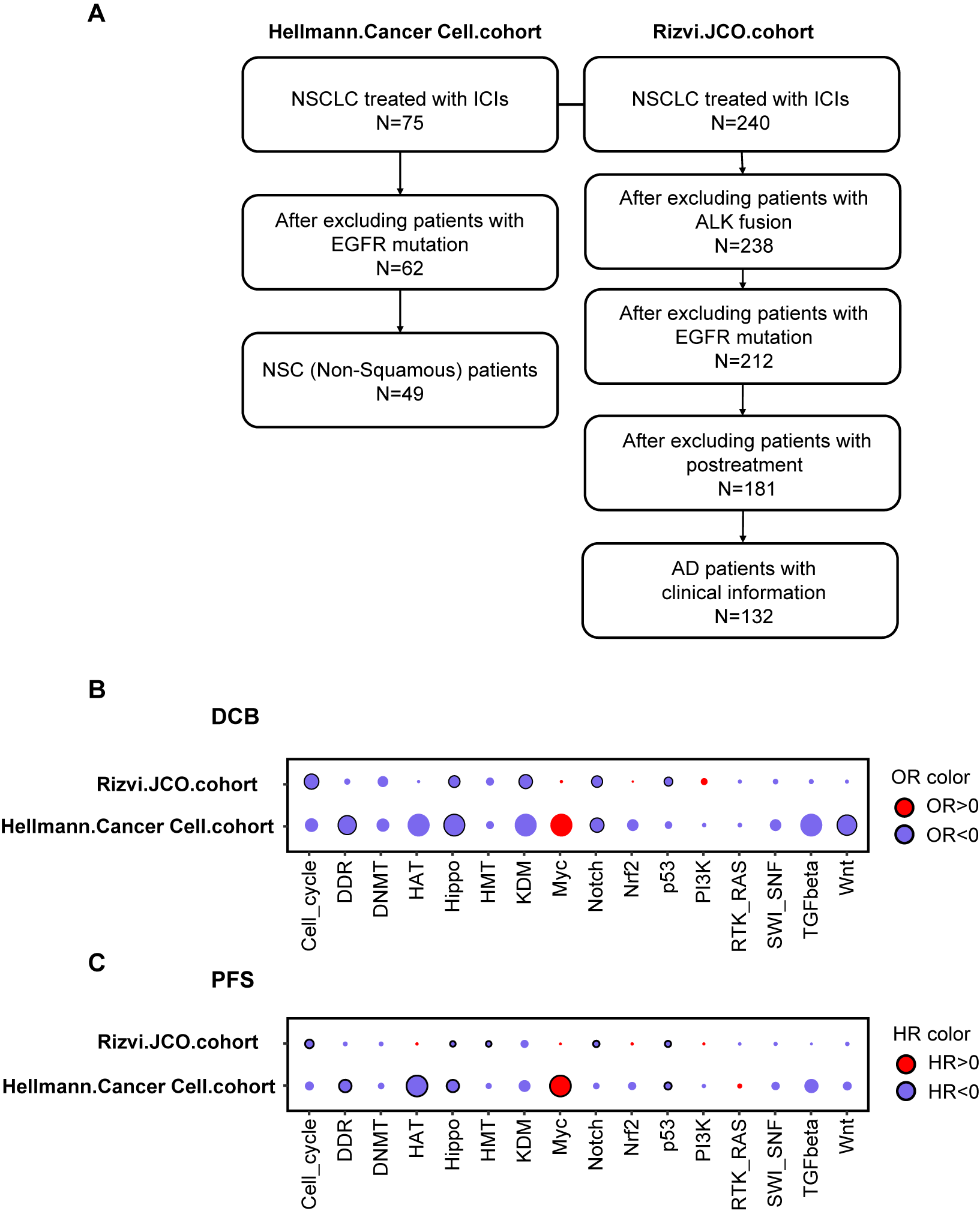

Supplement: Supplementary file 1 — Figure S1. [file CAM4-13-e7038-s004.tif]

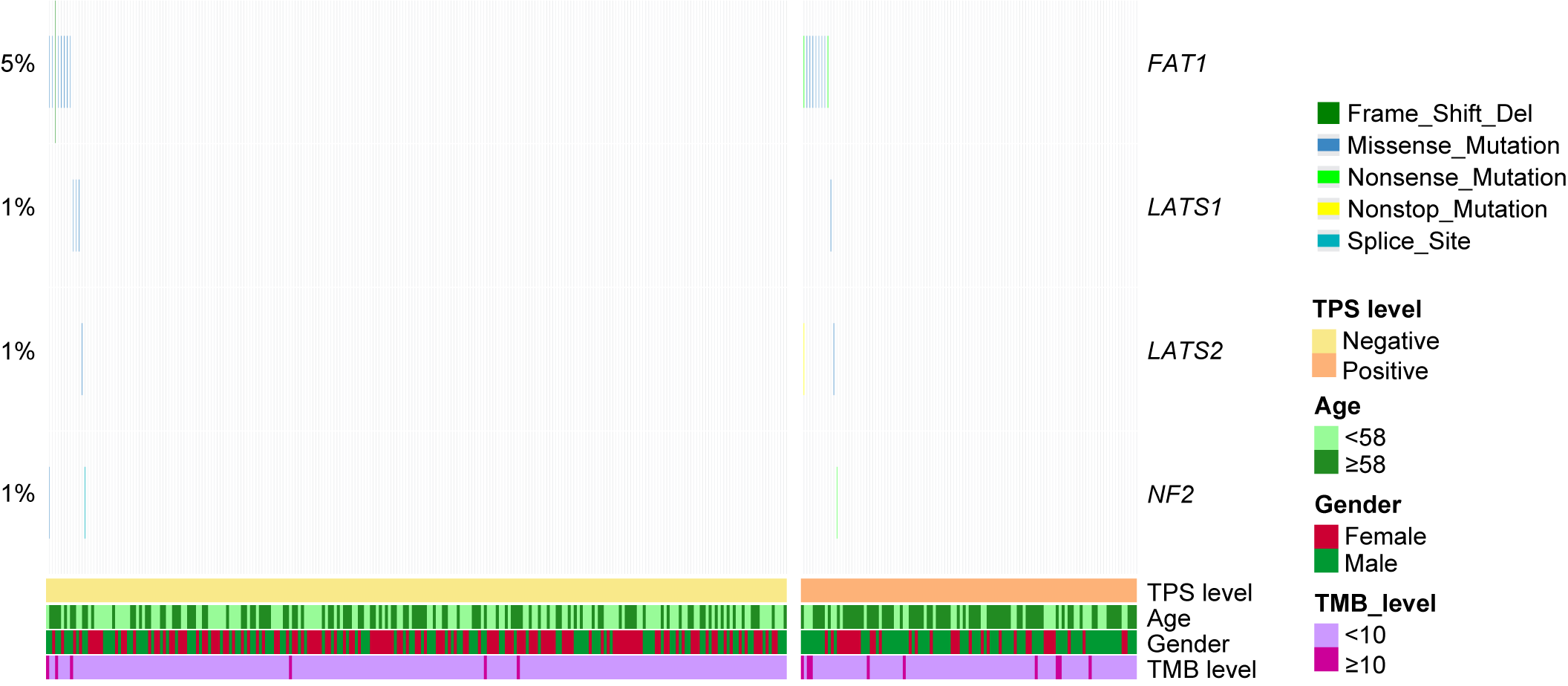

Supplement: Supplementary file 2 — Figure S2. [file CAM4-13-e7038-s003.tif]

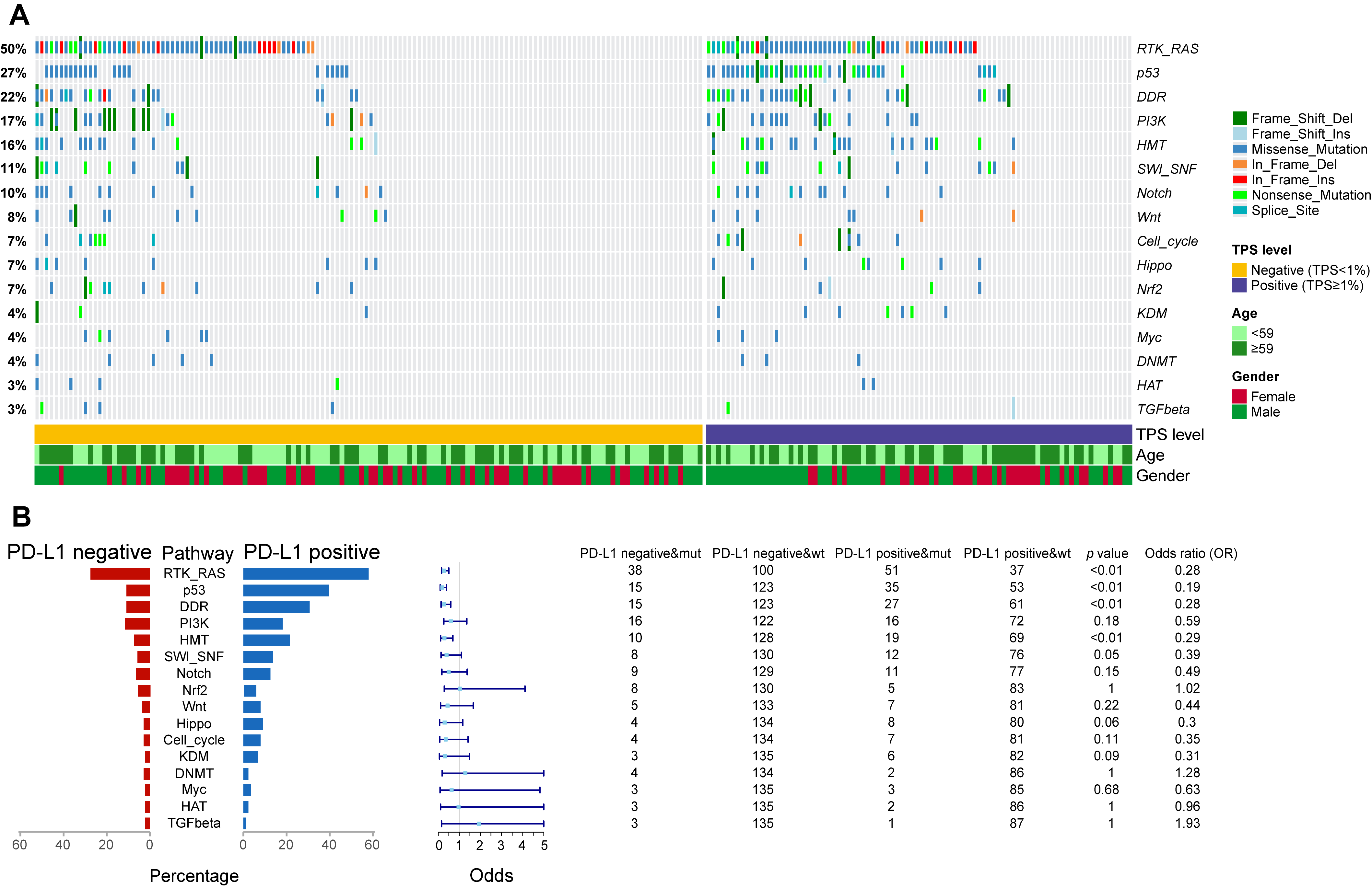

Supplement: Supplementary file 3 — Figure S3. [file CAM4-13-e7038-s005.tif]

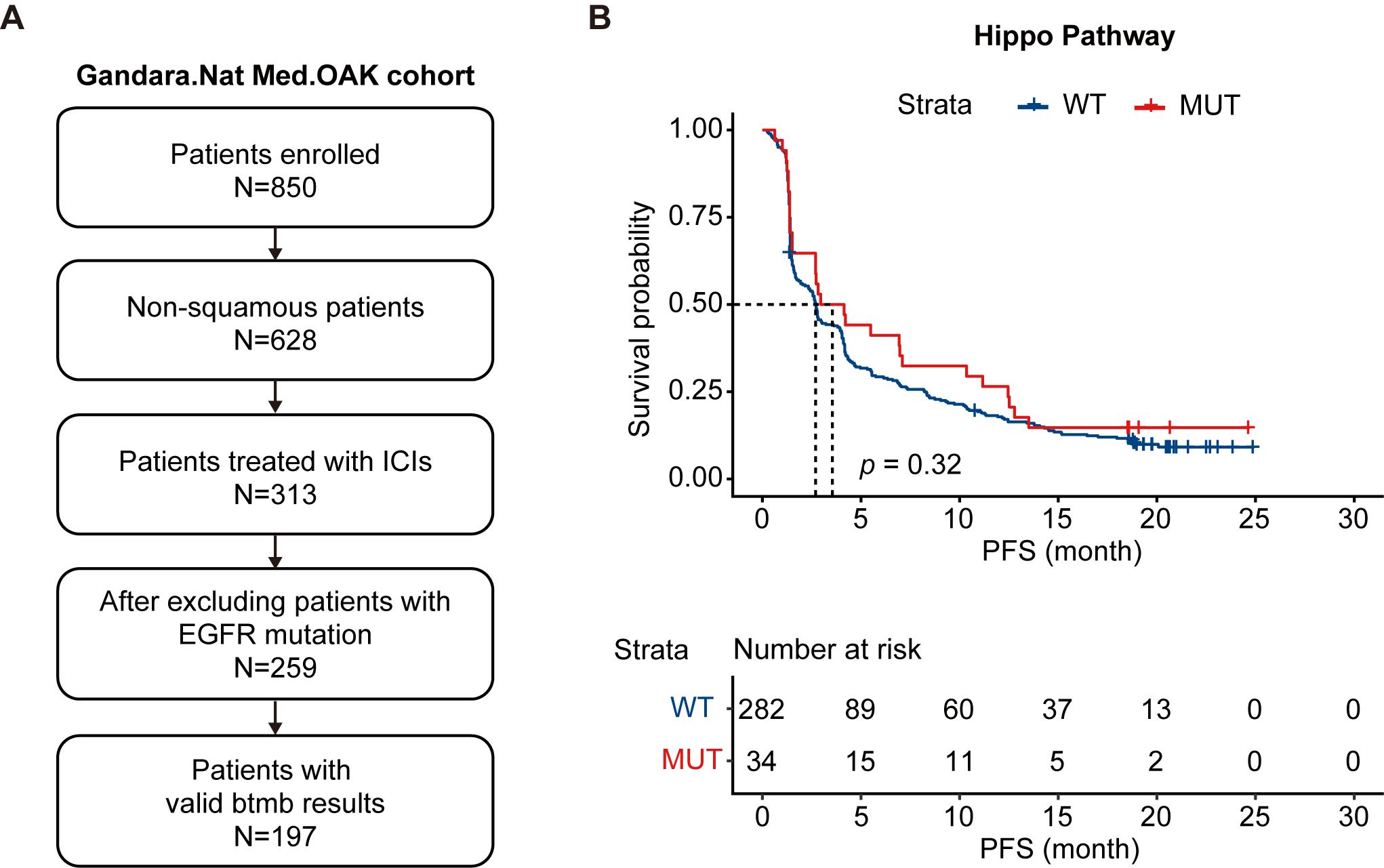

Supplement: Supplementary file 4 — Figure S4. [file CAM4-13-e7038-s002.tif]
