# Supplementary material for: The association of genomic alterations with PD‐L1 expression in Chinese patients with EGFR/ALK wild‐type lung adenocarcinoma and potential predictive value of Hippo pathway mutations to immunotherapy
Source: Cancer Med. 2024 Feb 23;13(3):e7038. doi: 10.1002/cam4.7038 (PMC10891359; doi:10.1002/cam4.7038)
Supplement: Supplementary file 8 — Table S4. [file CAM4-13-e7038-s001.docx]

| Variable | Overall,  N = 359^1^ | Negative (TPS<1%)  , N = 247^1^ | Positive (TPS≥1%)  , N = 112^1^ | p-value^2^ |
| --- | --- | --- | --- | --- |
| **Stage** |  |  |  | <0.001 |
| Ⅰ | 209 (58.22%) | 168 (68.02%) | 41 (36.61%) |  |
| Ⅱ | 50 (13.93%) | 37 (14.98%) | 13 (11.61%) |  |
| Ⅲ | 46 (12.81%) | 18 (7.29%) | 28 (25.00%) |  |
| Ⅳ | 54 (15.04%) | 24 (9.72%) | 30 (26.79%) |  |
| **Gender** |  |  |  | <0.001 |
| Female | 166 (46.24%) | 132 (53.44%) | 34 (30.36%) |  |
| Male | 193 (53.76%) | 115 (46.56%) | 78 (69.64%) |  |
| **Age** | 57.09 ± 12.08 | 55.22 ± 12.51 | 61.21 ± 9.97 | <0.001 |
| ^1^n (%); Mean (SD) | | | | |
| ^2^Fisher's exact test; Pearson's Chi-squared test; t-test | | | | |
