# Supplementary material for: The association of genomic alterations with PD‐L1 expression in Chinese patients with EGFR/ALK wild‐type lung adenocarcinoma and potential predictive value of Hippo pathway mutations to immunotherapy
Source: Cancer Med. 2024 Feb 23;13(3):e7038. doi: 10.1002/cam4.7038 (PMC10891359; doi:10.1002/cam4.7038)
Supplement: Supplementary file 9 — Table S5. [file CAM4-13-e7038-s006.docx]

| Variable | Before PSM | | | | | |  | After PSM | | | | | |
| --- | --- | --- | --- | --- | --- | --- | --- | --- | --- | --- | --- | --- | --- |
|  | Total (n = 359) | PD-L1  negative  (n = 247) | PD-L1  positive  (n = 112) | Statistic | P | SMD |  | Total (n = 226) | PD-L1  negative  (n = 138) | PD-L1  positive (n = 88) | Statistic | P | SMD |
| Age, Mean ± SD | 57.09 ± 12.08 | 55.22 ± 12.51 | 61.21 ± 9.97 | t=-4.863 | <0.001 | 0.602 |  | 59.20 ± 9.96 | 58.93 ± 10.20 | 59.61 ± 9.62 | t=-0.499 | 0.618 | 0.071 |
| Stage, n (%) |  |  |  | χ²=47.481 | <0.001 |  |  |  |  |  | χ²=6.658 | 0.152 |  |
| I | 209 (58.22%) | 168 (68.02%) | 41 (36.61%) |  |  | -0.669 |  | 114 (48.23) | 74 (52.17) | 40(42.05) |  |  | -0.205 |
| II | 50 (13.93%) | 37 (14.98%) | 13 (11.61%) |  |  | -0.105 |  | 36 (15.93) | 25 (18.12) | 11 (12.50) |  |  | -0.170 |
| III | 46 (12.81%) | 18 (7.29%) | 28 (25.00%) |  |  | 0.409 |  | 36 (15.93) | 17 (12.32) | 19 (21.59) |  |  | 0.225 |
| IV | 54 (15.04%) | 24 (9.72%) | 30 (26.79%) |  |  | 0.385 |  | 40 (17.7) | 22 (15.94) | 18 (20.45) |  |  | 0.112 |
| Gender, n (%) |  |  |  | χ²=16.519 | <0.001 |  |  |  |  |  | χ²=0.159 | 0.690 |  |
| Female | 166 (46.24) | 132 (53.44) | 34 (30.36) |  |  | -0.502 |  | 91 (40.27) | 57 (41.30) | 34 (38.64) |  |  | -0.055 |
| Male | 193 (53.76) | 115 (46.56) | 78 (69.64) |  |  | 0.502 |  | 135 (59.73) | 81 (58.70) | 54 (61.36) |  |  | 0.055 |
